# Role of Toll-Like Receptors in Actuating Stem/Progenitor Cell Repair Mechanisms: Different Functions in Different Cells

**DOI:** 10.1155/2019/6795845

**Published:** 2019-04-04

**Authors:** Fabio Sallustio, Claudia Curci, Alessandra Stasi, Giuseppe De Palma, Chiara Divella, Roberto Gramignoli, Giuseppe Castellano, Anna Gallone, Loreto Gesualdo

**Affiliations:** ^1^Department of Basic Medical Sciences, Neuroscience and Sense Organs, University of Bari “Aldo Moro”, Bari 70124, Italy; ^2^Nephrology Unit, Department of Emergency and Organ Transplantation, University of Bari “Aldo Moro”, Bari 70124, Italy; ^3^Institutional Biobank, Experimental Oncology and Biobank Management Unit, IRCCS Istituto Tumori “Giovanni Paolo II”, Bari, Italy; ^4^Department of Laboratory Medicine, Division of Pathology, Karolinska Institutet, SE-171 76 Stockholm, Sweden

## Abstract

Toll-like receptors (TLRs) represent one of the bridges that regulate the cross-talk between the innate and adaptive immune systems. TLRs interact with molecules shared and preserved by the pathogens of origin but also with endogenous molecules (damage/danger-associated molecular patterns (DAMPs)) that derive from injured tissues. This is probably why TLRs have been found to be expressed on several kinds of stem/progenitor cells (SCs). In these cells, the role of TLRs in the regulation of the basal motility, proliferation, differentiation processes, self-renewal, and immunomodulation has been demonstrated. In this review, we analyze the many different functions that the TLRs assume in SCs, pointing out that they can have different effects, depending on the background and on the kind of ligands that they recognize. Moreover, we discuss the TLR involvement in the response of SC to specific tissue damage and in the reparative processes, as well as how the identification of molecules mediating the differential function of TLR signaling could be decisive for the development of new therapeutic strategies. Considering the available studies on TLRs in SCs, here we address the importance of TLRs in sensing an injury by stem/progenitor cells and in determining their behavior and reparative activity, which is dependent on the conditions. Therefore, it could be conceivable that SCs employed in therapy could be potentially exposed to TLR ligands, which might modulate their therapeutic potential *in vivo*. In this context, to modulate SC proliferation, survival, migration, and differentiation in the pathological environment, we need to better understand the mechanisms of action of TLRs on SCs and learn how to control these receptors and their downstream pathways in a precise way. In this manner, in the future, cell therapy could be improved and made safer.

## 1. Introduction

Toll-like receptors (TLRs) are noncatalytic molecules with a single transmembrane segment. TLRs belong to the most extensive family of pattern recognition receptors (PRR) [1] and play a crucial role in innate defense against microorganisms and in the recognition and response to endogenous molecules that derive from injured tissues (damage/danger-associated molecular patterns, or DAMPs [1]). Although all classes of DAMPs can bind to TLRs and have some overlap in molecular machinery with PAMPs, there is evidence that DAMPs utilize different binding sites [2] and distinct mechanisms of action [3, 4]. Moreover, interesting findings demonstrated that there are also differences in the downstream TLR signaling [5] and subsequent biological outcomes [3, 4]. TLRs have been named after the identification of the *TOLL* gene in Drosophila more than 30 years ago, and within the past three decades, 13 different types of human TLRs have been identified, as have many polymorphic forms in several other mammalian species. Commonly, Toll-like receptors have been subdivided into two groups based on cellular localization: TLR1, TLR2, TLR4, TLR5, TLR6, TLR10, TLR11, TLR12, and TLR13 are typically expressed on the cell surface; TLR3, TLR7, TLR8, and TLR9 localized mainly on intracellular endosomes.

Their expression throughout both animal and plant kingdoms [6–8] highlights their important role in physiological and pathological conditions. TLRs recognize a wide range of structurally conserved molecules commonly expressed on bacterial, viral, and fungal surfaces, collectively grouped as pathogen-associated molecular patterns (PAMPs) [9, 10]. PAMP molecules interact with pattern recognition molecules (PRMs) on the surface of immune cells [11]. TLRs are included within PRMs. As opposed to PRRs, the term PRMs has been used to refer to a more broad group of components of the innate system, which include secreted molecules that bind to microorganisms [12].

TLRs have been localized on macrophage, neutrophil, dendritic, and NK cells. Following recognition, TLR activates the immune response; indeed, the interaction between TLR and PAMP leads to a typical inflammatory response characterized by a cascade of intracellular signals [1, 6, 10, 13]. Nonetheless, TLRs are also involved in antigen presentation and process, accentuating their key role in regulating the cross-talk between innate and adaptive immune responses [10, 14–16].

In addition to several cells of the immune system, TLRs have been found on several kinds of stem/progenitor cells (SC). In such cells, the role of TLR has been ascribed to basal motility, self-renewal, differentiation potential, and immunomodulation. In this review, we will describe several different functions that TLR carries out in SC, focusing on SC's plastic role in response to specific ligands. Moreover, TLR has been shown to take over important functions during the reparative processes carried out by the SCs, consistent with the TLR dependence for the correct establishment of dorsoventral patterning during development in Drosophila [17]. We intend to describe and discuss the role played by TLRs in such reparative processes performed by different tissue stem/progenitor cells, with a specific interest in new therapeutic strategies.

## 2. TLRs and Mesenchymal Stromal Cells

Since their first description more than 30 years ago, mesenchymal stromal cells (MSCs) have been identified in essentially all the tissues of the human body, with a major source of cells for clinical uses in bone marrow (BM-MSC), adipose tissue (AT-MSC), and perinatal tissues as placenta or umbilical cord (Wharton jelly (WJ-MSC) or umbilical cord blood (UCB-MSC)) [18, 19]. MSC is a term coined by Caplan [18] during the first clinical applications, and since then autologous BM- and AT-derived MSCs have been the most extensively studied sources of stromal cells. Often misinterpreted, indicated as mesenchymal stem cells instead of stromal cells, adult and perinatal MSCs have recently led to evidence supporting similar, but not identical, behavior and properties in most if not all the human MSCs [20, 21].

MSCs have been shown to express high levels of TLRs, broadly distributed on the cell surface. TLRs have been proposed to modulate MSC proliferative, immunomodulatory, and migratory and differentiation potential [19, 22]. Several studies have measured the expression and transduction pattern of TLR in MSCs from different sources, with BM-MSC once more as the most characterized source, while limited evidence has been collected so far on adipose tissue and umbilical cord, sometimes with opposite results [23].

Nowadays, it is well-accepted that human BM-MSC, AT-MSC, and UCB-MSC express high levels of TLR3 and TLR4, in addition to low levels of TLR1, TLR2, TLR5, TLR6, and TLR9 [24]. Lack of expression in TLR7, TLR8, and TLR10 forms has been reported in almost all the MSCs examined. Interestingly, a limited expression of the TLR3 isoform has been described on WJ-MSC, and such receptors appear to be nonfunctional as its ligation did not release TLR-inducible cytokines. Such peculiar expression has been coupled with a low immunogenic phenotype and inefficient response to LPS activation by WJ-MSC [19, 25].

The expression of TLR on MSC prompted researchers to investigate the potential link between TLR signaling and MSC anti-inflammatory and immune-modulatory properties [26].

### 2.1. TLRs in Immunomodulatory Properties of MSC

MSCs possess immunoregulatory properties which have been extensively characterized for their relevance in immune responses and exploited in clinical applications. Human MSCs can alter inflammatory conditions and might influence different effector cells, moving from lymphoid cells (T, B, and NK cells), to myeloid components (monocytes, dendritic cells) [27–30]. The MSC effect has been ascribed mainly to cell-to-cell contact and the release of soluble factors, such as transforming growth factor- (TGF-) *β*1, hepatocyte growth factor (HGF), prostaglandin E2, interleukin- (IL-) 10, indoleamine 2,3-dioxygenase (IDO), interferon- (IFN-) *γ*, and nitric oxide (NO), upon activation in response to inflammation [22, 24, 27].

**Figure 1:**
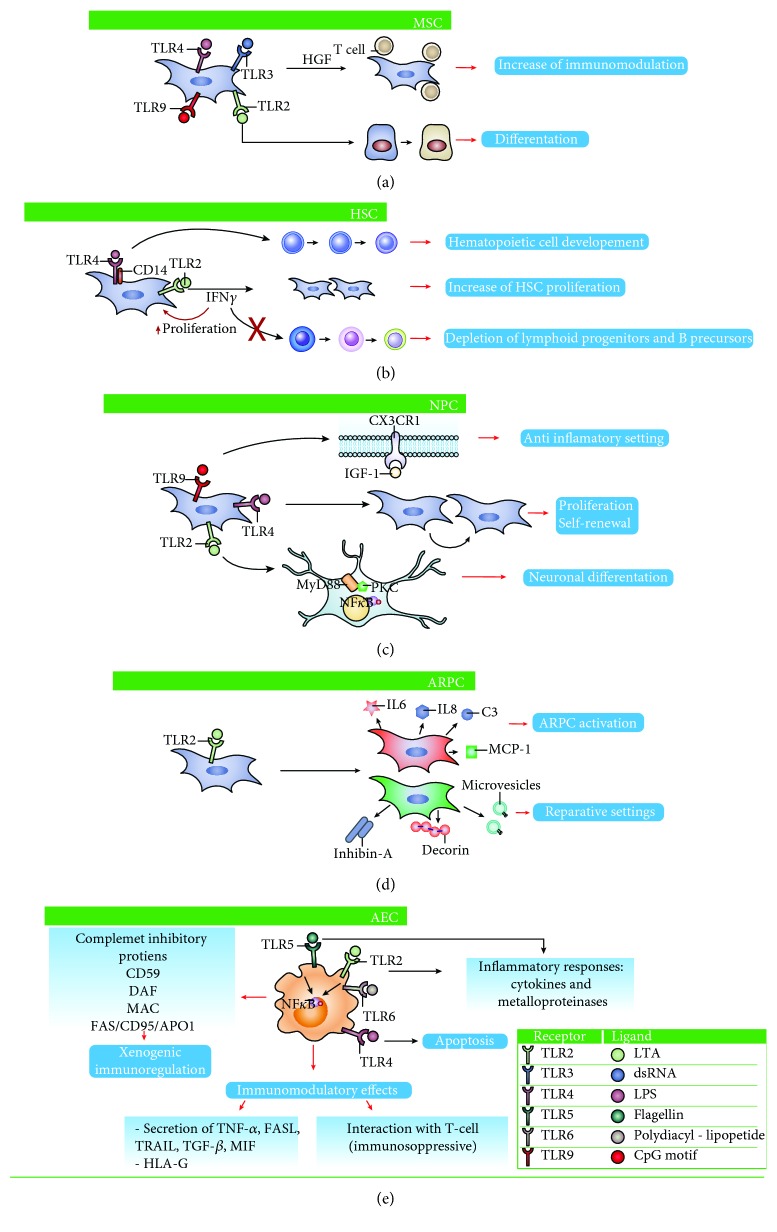
Comparison of biological functions of TLRs on stem cells. (a) In MSCs, TLR3 and TLR4 triggering induces an immunomodulation increase, while TLR2 is involved in differentiation processes. (b) TLR4 and TLR2 play a major role in influencing the cell biology of HSCs. TLR4 triggers hematopoietic cell development. TLR2 induces an increase in HSC proliferation, avoiding depletion of lymphoid progenitors and B cell precursors. (c) Both TLR2 and TLR4 have distinct and even opposite functions, concerning the self-renewal, proliferation, and differentiation of NPC. The activation of TLR9 leads to neuroprotective effects by anti-inflammatory mechanisms. (d) Among TLRs, TLR2 is strongly upregulated in ARPCs, and it is principally involved in reparative properties of ARPCs. TLR2 is responsible for the secretion of several reparative cytokines and chemokines, including IL-6, IL-8, C3, MCP-1, inhibin-A, and decorin. (e) AEC expresses various TLR family members and responds to multiple TLRs ligands that heavily influence the cell behavior.

The antiproliferative effect on T cells has been widely studied, showing different and sometimes contrasting results. AT-MSCs do not constitutively express immunomodulatory factors, yet releasing inhibitory factors upon activation [31]. Moreover, TLR activation does not affect the AT-MSC immunomodulatory properties [32]. Almost 10 years ago, a study showed how TLR3 and TLR4 ligation enhances the immunomodulatory properties in BM-MSC [28] (Figure 1(a)). Notch signaling and upregulation of delta-like 1 (DL1) have been shown to enhance Treg induction driven by TLR3- and TLR4-activated MSCs [33]. In contrast, another study showed how ligation of TLR3 or TLR4 on BM-MSC negatively affects T-cell proliferation inhibition by hampering T cell Jagged-1 expression and, therefore, impairing its signaling to the Notch receptor [27]. Such opposite effects have been explained by the influence of the inflammatory environment exercised on cells. Short-term, low-level exposure with TLR4 agonists polarizes MSCs toward a proinflammatory phenotype, critical for early injury responses. TLR4-primed MSC results in collagen deposition, expression of proinflammatory mediators, and reversal of the T-cell suppressive mechanisms. By contrast, TLR3 agonist exposure has appeared to prompt MSC committing toward an immunosuppressive phenotype, critical to anti-inflammatory reactions that assist with resolving the tissue damage. In addition to TLR3, stimulation of TLR9 by DSP30 (a CpG ODN) was also shown to induce proliferation and the suppressive potential of BM-MSC, protecting them from TLR4 stimulation by LPS, which restricted the ability of MSC to suppress the proliferation of T lymphocytes [34]. Therefore, MSCs have been described as switching toward pro- or anti-inflammatory phenotypes, depending on which TLR forms (TLR3 or TLR4) are expressed on their surface or which ligands they can sense [35]. Another characteristic response to TLR ligands has been described on WJ-MSCs, which do not respond to TLR4 or TLR3 ligation. Such an effect might rely on the overexpression of factors involved in immune system modulation (i.e., HGF) or on the expression of nonfunctional TLR [19].

Together, these data represent a serious warning for clinical use of MSCs. Their immunomodulatory aptitude represents a key factor for therapeutic application [31]. The beneficial contribution of MSCs might be diminished or erased if inflammation is present [22]. Consequently, WJ-MSCs, with their aforementioned TLR3 limited expression, represent an attractive source of cells with proficient immunomodulatory properties [24]. Although silencing some TLR forms might represent an effective way to maximize the immunomodulatory effect of several MSCs, the molecular mechanisms and effects on TLR-primed MSCs need to be elucidated before moving to the bedside.

### 2.2. TLRs in Differentiation Capacity of MSC

The differentiation capacity of different MSCs towards multiple tissue phenotypes has been largely mentioned and often described as an age-dependent mechanism [24]. However, recent studies highlight the important role played by TLR molecules in MSC maturation into different cell phenotypes. The activation of TLRs has been shown to influence MSC maturation into osteocytes. Osteoblastic maturation has been described by a specific agonist triggering TLR2, TLR3, and TLR4 activation [27, 32, 36–38]. Moreover, the activation of TLR-9 by CpG oligodeoxynucleotides (CpG-ODN) can reduce AT-MSC proliferation and enhance osteocyte differentiation [37] (Figure 1(a)). The TLR9 agonist CpG oligodeoxynucleotide (CpG-ODN) with a phosphorothioate backbone (PTO-CpG-ODN) has been described to antagonize BMP-induced Smad signaling in a TLR9-independent manner, thus inhibiting osteoblast maturation by AT- and UCB-MSCs [31]. Furthermore, during osteogenic differentiation, TLR9 expression has been shown to be significantly decreased [26]. Differently, LPS (TLR4 agonist) or flagellin (TLR5 agonist) has been shown to trigger osteogenic differentiation in UCB-MSCs [39]. However, these data need to be confirmed by more rigorous studies.

There are no data supporting a role for TLR in adipogenic differentiation. Notably, few and contradictory reports support the role of TLR2 in chondrogenic maturation, underlying a need to deepen this specific feature of MSC biology.

### 2.3. TLRs in Migration of MSCs

MSCs also have the important capacity to transfer to the places of ischemic, inflammatory, or mechanical damage or to the site of tumor growth [40].

The effect of TLR stimulation on MSC migration has been examined using different TLR agonists as chemoattractants. The results showed TLR3 as the main mediator in migration responses [41]. However, this effect seems to be strongly related to the time of exposure: after 1 hour of incubation, both TLR3 and TLR4 promoted migration, while 24 h incubation with the same TLR chemoattractants suppressed migration and invasion of the treated MSCs [27, 35]. Moreover, the inhibition of TLR3 and TLR4 expression with knockdown plasmids cut in half the migration potential of unprimed MSCs [41]. Nevertheless, LPS or poly(I:C) treatment of the transfected cells resulted in enhanced migration when compared with unstimulated controls [35]. In addition to the role played by TLR3 and TLR4, TLR9 activation has also been shown to facilitate MSC migration towards target tissues, in an MMP-13-mediated mechanism [26].

The different responses of MSCs, depending on the even minimal changes in the environment, again support the TLR regulation of these cells by complex and mostly unknown molecular mechanisms.

## 3. TLRs and Dental Mesenchymal Stem Cells

Mesenchymal stem cell populations with high proliferative capacity and multilineage differentiation have been isolated from the dental tissues. These are dental pulp stem cells (DPSCs), stem cells from human exfoliated deciduous teeth (SHEDs), periodontal ligament stem cells (PDLSCs), dental follicle progenitor stem cells (DFPCs), and stem cells from apical papilla (SCAPs). DPSCs and SHEDs are characterized by the expression of markers for both mesenchymal and neuroectodermal stem cells and derive from the cranial neural crest. DPSCs can differentiate into several cell types including odontoblasts, neural progenitors, chondrocytes, endotheliocytes, adipocytes, smooth muscle cells, and osteoblasts [42].

To date, the role of TLRs linked to the regenerative properties of dental stem cells has not been reported. Few studies have explored TLR expression profiles in dental stem cells. In an uninflamed environment, DPSCs expressed high levels of TLR10, followed by TLR2, TLR1, TLR5, TLR4, TLR9, TLR7, TLR6, TLR3, and TLR8 in descending order of expression. The inflammatory environment upregulated TLR2, TLR3, TLR4, TLR5, and TLR8; downregulated TLR1, TLR7, TLR9, and TLR10; and abolished TLR6 expression in DPSCs [43].

TLR4's role in regulating immunomodulation or osteogenic capacity in some kind of dental mesenchymal stem cells has been shown. In fact, during neuroinflammation, in neurodegenerative diseases, TLR4 in DPSCs can induce the secretion of soluble factors, such as interleukin-8, interleukin-6, and TGF-*β*-enhancing cell immunomodulatory properties [44]. *In vitro*, LPS can activate the TLR4-regulated NF-*κ*B pathway of human PDLSCs, thus decreasing their osteogenic potential. This potential can be reverted by impeding the TLR4 binding or neutralizing the NF-*κ*B pathway, thereby avoiding bone loss triggered by LPS in rats [45].

## 4. TLRs and Hematopoietic Stem Cells

Of all stem cells, hematopoietic stem cells (HSCs) are by far the most studied and infused in patients with cancers, such as multiple myeloma or leukemia. Since HSCs represent the capstone of the blood hierarchy, they can reconstitute the entire hematolymphoid system, making them a powerful tool for blood disorders [46].

Early HSCs expressed functional TLR2 and TLR4. During infection, microbial components could activate quiescent stem cells through TLR signaling promoting myeloid differentiation and rapidly replenishing the innate immune system. Signaling in granulocyte and macrophage progenitors through Myd88 downstream TLR2 and TLR4 eliminates the need for growth and differentiation factors. LPS were efficaciously recognized in HSC by the TLR4/MD-2 complex interacting with the CD14 coreceptor. Moreover, common lymphoid progenitors are preferentially directed toward dendritic cell differentiation [47] (Figure 1(b)). Interestingly, in response to TLR ligand stimulation, murine short-term HSC has been proved more efficient in producing cytokines than mature immune cells [48].

TLR expression on myeloid cells has been shown to sense bacterial products, inducing myelopoiesis. HSCs have been shown to be activated by LPS exposure, either directly through cell-intrinsic TLR signaling or indirectly through the upregulation of myeloid-derived inflammatory cytokines. *In vivo* chronic treatment with LPS leads to HSC cycling and to myeloid differentiation with a consequent loss of their repopulating activity in transplantation experiments [49, 50]. Indeed, the TLR4/Sca-1 axis contributes to granulopoiesis starting from HSC during bacterial infection or LPS treatment [51]. Notably, the time and the entity of the stimulation can influence cell lymphopoiesis as proved by chronic low-dose LPS perturbation in human HSC and B-lineage progenitors. The increased amount of proliferating HSC couples with a higher level of IFN-*γ* protein, suggesting a potential local source of this cytokine. This leads to a depletion of lymphoid progenitors and B precursors [52] (Figure 1(b)). LPS treatment increases demand for myeloid cells and specifically employs myeloid-biased HSCs (MB-HSCs) and progenitors into the cell cycle. In addition to LPS receptor TLR4, histamine also plays an important role in HSC expansion, hampering cycling MB-HSC depletion [53]. LPS stimulation *in vivo* induces proliferation of HSC directly through TLR4 interaction; however, prolonged LPS exposure weakens HSC self-renewal and repopulation activity. Therefore, while initial TLR4 activation in HSC might be advantageous to counteract systemic infection, protracted TLR4 signaling might have deleterious effects and lead to inflammation-related dysfunction [54]. However, systemic exposure to the TLR2 agonist leads to a loss of HSC self-renewal in bone marrow. Such effects have been shown, at least in part, to be mediated by the granulocyte colony-stimulating factor and tumor necrosis factor-*α* [55].

In conclusion, these studies support a mechanism mediated by TLR signaling, in which HSCs sense non-self PAMPs, allowing them to rapidly respond to infections in order to replenish the hematopoietic system; however, prolonged exposure may affect self-renewal and differentiation leading to HSC pool exhaustion.

## 5. TLRs and Neuronal Stem/Progenitor Cells

In the adult brain, TLRs regulate neurogenesis, as shown in the murine hippocampus. TLRs may also have a role in the protection of neurons, by favoring remyelination and trophic support [56]. It has been shown that the TLR3 form triggers secretion of anti-inflammatory cytokines as IL-9, IL-10, and IL-11, with an inhibitory effect on astrocyte growth, and enhanced neuronal survival [57].

Moreover, neuronal stem/progenitor cells (NPC) are responsive to TLR3 stimulation with poly(I:C), secreting proinflammatory cytokine as IL-6 but not TNF-a, whereas the microglia are responsive to ligands of both TLR3 and TLR4 (through poly(I:C) and LPS exposure, respectively) [58]. The TLR2, TLR3, and TLR4 forms have been proven to inhibit NPC cell proliferation when exposed to specific ligands [58–60].

TLR2 and TLR4 are abundant isoforms in the central nervous system [61, 62], and their presence extends to the neurogenic niche rich in adult stem/progenitor cells. Both TLR2 and TLR4 have been identified on adult NPC with distinct and sometimes opposite functions in proliferation and neuronal differentiation. The distinct effects played by the two TLR receptors, normally present on the same neuronal cell, suggested a specific action yet not completely elucidated. The absence of TLR2 has been associated with delays in neuronal differentiation, but with no direct effect on proliferative rhythm. On the contrary, lack of TLR4 leads to an increased rate in proliferation and differentiation [63]. Moreover, *in vivo*, TLR2 knockout mice have been shown to have extremely hindered neuronal differentiation, with preferred astrocytic maturation [63]. These data together suggest that TLR2 might play a direct role in neuronal stem cell maturation, as confirmed by wild-type mice where neuronal differentiation was increased using increasing doses of TLR2 activators [63]. The different effect produced by the two TLRs could be explained by the activation of a dissimilar transduction pathway. TLR2 activation leads to MyD88-mediated activation of NF-*κ*B pathways with the contribution of PKC-alpha. The inhibition of PKC kinase in the presence of TLR2 activators leads to reduced differentiation capacity. On the other hand, inhibiting NF-*κ*B in the presence or absence of TLR2 ligands results in a drastic decrease in neuronal maturation [63] (Figure 1(c)).

Unlike TLR2, TLR4 can exert its effects through an independent MyD88 pathway, with a reported delay in NF-KB activation [6]. The activation of TLR4, by ultrapure LPS formulation, significantly decreases the neurodifferentiation process; on the contrary, TLR4 silencing promotes neural stem cell differentiation. In addition to the different molecular adapters, there are also differences concerning the timing of activation of NF-KB, which is delayed in the case of the independent MyD88 pathway [6] (Figure 1(c)). Instead, in both cases, there are no differences regarding cell survival.

The distinct effects of the two receptors, constitutively present on the same neuronal cell, have been proved, with a predominant role played by TLR4 in neuronal self-renewing and differentiating power [64]. Such results suggest that TLR2 might behave as an antagonist towards TLR4, attenuating its effect on differentiation. Furthermore, by inhibiting MyD88, common to both molecular pathways, there is an increase in proliferation and differentiation, effects similar to those seen in the absence of TLR4. Notably, several TLR forms have been identified on the surface of immune cells and astrocytes, exclusively in the neurogenic niche [65, 66].

The contributions of the different cells expressing TLRs might differ depending on the physiological or pathological conditions.

In the setting of neural development, recent evidence supports an important role in cellular proliferation, differentiation, and survival/migration at different developmental phases for additional TLR isoforms. Indeed, TLR8 has been found to suppress neurite outgrowth and to induce neuronal apoptosis by means of a NF-*κ*B-independent mechanism [67].

Finally, TLRs have a role in the regeneration or neuroprotective effect exerted by NPCs. Thus, TLR9 stimulation by CpG oligodeoxynucleotides (ODN) has been shown to produce the secretion of neuroprotective molecules, such as CX3CR1 and insulin growth factor 1, and to the activation of the TLR9-ERK1/2 pathway. In such context, CpG-ODN might prompt NPC to direct microglia towards a beneficial phenotype through the release of diffusible factors and to switch microglia from a proinflammatory to an anti-inflammatory setting [68] (Figure 1(c)).

These observations further underline the importance of TLRs, according to their specificity to bind various stimuli, to trigger the NPC response under physiological and pathological conditions.

## 6. TLRs and Renal Stem/Progenitor Cells

Resident adult renal progenitor cells (ARPCs) have been recently isolated from both tubules and glomeruli of the human kidney. These two cell populations share surface markers, CD24, CD133, and Pax2, a transcription factor found in undifferentiated mesenchyme, and their gene expression profiles are similar [69–73]. To date, studies by other groups [73–76] and our research group [69, 77–79] suggest that both tubular and glomerular ARPCs could be an alternative source for the cellular therapy in kidney diseases for their multipotent differentiation ability and for their reparative properties [74, 75, 77, 79]. Once injected into acute or chronic renal injury models, these cells have been shown to regenerate tubular cells and improve renal function [70, 71, 73, 76]. Additional studies support the contribution of ARPCs in repairing injured renal parenchyma in patients with acute or chronic tubular damage [80].

Several publications have demonstrated the expression of TLRs in several tissues, but the importance of these receptors in ARPCs is novel. Since the TLRs respond to PAMPs and DAMPs, initial studies focused on TLR expression and function in renal tissue. Leemans et al. elucidated the role of TLR2 in chronic renal injury which is characterized by inflammation, apoptosis, and fibrosis [81]. They found that TLR2 is involved in the renal inflammatory response in the first phase of obstructive nephropathy, but not in the development of renal fibrosis and in subsequent progressive injury [81]. For the first time, our group showed that TLR2 is upregulated in ARPCs and it is responsible for their activation promoting the renal repair after kidney injury [69]. TLR2 might serve as a tissue damage sensor. Indeed, ARPCs secrete MCP-1 and C3, via NF-*κ*B activation, in response to TLR2 stimulation, as well as proinflammatory cytokines (IL-6 and IL-8) [69] (Figure 1(d)). The production of these cytokines and chemokines can be useful for the renal repair processes, as supported by preclinical experiments in a rat model of glycerol-induced acute kidney injury, where IL-6 has been shown to induce tubular regeneration and protect from further injuries [82, 83]. The cleavage fragments of C3, IL-8, and MCP-1 play important roles in mobilizing SC and modulate their trafficking [77, 84]. Moreover, upon TLR2 stimulation, ARPCs increased their proliferation rate in order to augment the pool of resident cells and prevent depletion [69].

In addition, TLR2 activation on resident tARPCs induces reparative processes by avoiding cisplatin-induced apoptosis in renal proximal tubular epithelial cells (RPTECs). Tubular ARPCs, after RPTEC damage and upon TLR2 activation, have been shown to produce and secrete inhibin A and decorin (both as protein and as mRNA shuttled by microvesicles) involved in the tubular cell regenerative process. All these regenerative processes can be null in the presence of TLR2-blocking agents. Interestingly, glomerular ARPCs have been shown unable to induce tubular cell regeneration in similar preclinical settings [79].

These data highlight the importance of TLR2 in mediating the reparative properties of tARPCs (Figure 1(d)). Finally, TLR2 overexpression in ARPCs can be mediated by miRNAs. miRNAs are important regulators of stem cell fate and behavior and regulate many target genes. Among several miRNAs differentially modulated in tARPCs relative to RPTECs, the low level of miR-1225-5p has been shown to induce high TLR2 expression and regulate other important genes, such as PAX-8, IL-8, BMPR2, IGF1, inhibin-A, cyclin D1, and WNT1, all involved in ARPC regenerative processes [69, 85, 86].

Together, results of Leemans and our group support the use of ARPCs in the treatment of renal failure. However, the TLR efficiency in sensing an injury and in determining stem/progenitor cell behavior and reparative activity depends on the conditions in which cells are located.

## 7. TLRs and Placental Stem Cells

Toll-like receptors are also widely expressed in perinatal tissues, and particularly in the placenta. TLR's presence on trophoblasts, decidual cells, and the amniotic epithelium has been measured and linked to specific functions at the maternal-fetal interface [87, 88].

The expression of different TLR forms is characterized by a temporal and spatial manner. For example, TLR6 is not expressed during the first trimester, while a late gestational fetus has been shown to be positive for its expression [89]. TLR2 and TLR4 are constitutively expressed by villous cytotrophoblast and extravillous trophoblast, but not by syncytiotrophoblast (which will form the outer trophoblast layer). Such temporal expression allows placental tissues of fetal origin (such as amnion membrane) to perform a punctual response to microbial contamination that might happen during the 9 months of human pregnancy. In contrast to fetal tissue, very little is known about the expression of TLRs in the maternal decidua. Recent studies demonstrated that TLR4 is expressed by amniotic epithelial cells (hAEC), suggesting their key role in preserving amniotic fluid sterility [90]. Interestingly, soluble TLR2 forms have been found in amniotic fluid, interfering with the binding of the respective ligand to TLR2 and downregulating the host inflammatory response to bacteria. Altogether, these pieces of evidence underline the importance of the TLR system as a sentinel for a wide range of pathogens that might trigger the inflammatory response in amniotic fluid [88].

The placenta-derived stem cells have been suggested as an important therapeutical strategy in regenerative medicine due to their easy isolation, cellular multipotency, low immune response, and immunomodulatory capacities, as well as the lack of ethical issue [91, 92]. Fetal-origin placental cells have been commonly divided into four populations: hAEC and amniotic mesenchymal stromal cells (hAMSC) isolated from the amnion membrane, human chorionic mesenchymal stromal cells (hCMSC), and human chorionic trophoblastic cells (hCTC) from chorion and decidua, respectively [91–93].

The presence of TLR4 in hAMSC and its role in preterm premature rupture of the membrane in response to fetal fibronectin have been recently illustrated [94]. Another interesting study showed the expression of TLRs in hAMSC, with a particular interest in immune surveillance during infection and in eliciting a proinflammatory response upon TLR2 and TLR6 activation [95]. Such initial results suggest hAMSC and its role in immunomodulation during pregnancy.

Amniotic epithelial cells express several TLR family members (TLR5 and TLR6/2 are expressed and functionally active) and respond to multiple TLR ligands [96]. After stimulation with TLR6/2 and TRL5 agonists, hAECs produce and secrete proinflammatory cytokines, metalloproteinases (MMP-9), and activate the NF-*κ*B signaling pathway [92, 93, 96]. By contrast, TLR4 induction does not result in an inflammatory response but does activate apoptotic processes, which can lead to preterm premature rupture of membranes [96] (Figure 1(e)). It has been proposed that hAEC's response in the presence of intrauterine infection depends upon which TLR is activated [96]. However, further investigations are necessary to determine the role played by hAEC in the immune response and their importance as sentinels for a wide range of pathogens.

Similar to MSCs, hAECs have been reported to have immunomodulatory and anti-inflammatory properties that might be of particular benefit in regenerative medicine after an insult [97–100]. The expression of complement inhibitory proteins, CD59 antigen (decay-accelerating factor), membrane attack complex, and Fas antigen/CD95/APO1 has been shown to have significant effects in xenogeneic immunoregulation [97] (Figure 1(d)). Altogether, the expression of TLR in hAEC and their immunomodulatory properties suggest that these cells have the ability to correct inflammatory disease, and thus this approach has been proposed as the first allogeneic cell therapy that may not require supporting immunosuppression therapy [101]. The promising results obtained in recent preclinical studies for correction of liver diseases [92, 97, 98, 102] have suggested the use of hAEC in several acute and chronic disorders, not only liver-related.

## 8. TLRs and Intestinal Stem Cells

Intestinal stem cells (ISCs) reside at the base of the crypt region of the intestinal epithelium and have both the self-renewal capacity and the potential to differentiate into different cell types as Paneth cells, absorptive enterocytes, goblet cells, and enteroendocrine lineages [103].

The regulatory mechanisms that control stem cell proliferation in normal conditions and in response to injury are just beginning to be explored. When ISCs replicate by overcoming the normal controls of cell division, they can result in cancer; thus, maintaining a balance between self-renewal and differentiation of ISCs is a hallmark of an intestinal functional niche. An increasing number of signaling pathways, including Wnt, BMP, Hedgehog, and Notch, may play important roles in regulating stem cell proliferation [104].

To date, factors regulating the proliferation and apoptosis of ISCs remain incompletely understood. Because ISCs are in contact with microbial ligands, immune receptors such as Toll-like receptors could play a critical role [105]. In particular, during enterocolitis, overstressed TLR-4 repressed ISC proliferation and induced apoptosis through p53 upregulated modulator of apoptosis (PUMA). Therefore, the TLR4-PUMA axis might be a therapeutic target for this disorder [105]. It was also observed that putative human colonic stem cells express TLR-2, TLR-4, and TLR-5. In these cells, TLR-4 regulated Wnt signaling that controls stem cell function [106].

However, little is known about the effects of microbiota and TLR signaling on ISCs which may influence regeneration and protection of the damaged mucosal barrier [107, 108]. Recent studies demonstrated the protective effect of Lactobacillus reuteri D8 on the integrity of intestinal mucosa [108]. In particular, this lactobacillus caused the IL-22 release by lymphocytes of the lamina propria inducing ISC proliferation and promoting the intestinal epithelium recovery after a damage caused by TNF-*α* [108]. Moreover, even if the cross-talk among the entire microbiota and the TLR/MyD88 signaling on the ISCs is not yet well elucidated, it has been shown that MyD88−/− mice are more susceptible to acute dextran sodium sulfate- (DSS-) induced colitis and develop a more severe disease [109].

In addition, LPS (the TLR-4 agonist) is found in the crypt-specific core microbiota and can regulate intestinal epithelium proliferation by inducing death of stem cells by necroptosis and enhancing cell differentiation toward the goblet cell lineage. Besides, low and nontoxic concentrations of LPS increase the resistance to tissue damage after transplantation, improving parenchymal regeneration. Therefore, TLR-4 could have a great impact in modulating stem cell activity after intestinal transplantation [110].

TLR4 signaling could also be implicated in response to hypoxic stimulation, inducing ISC proliferation. Hypoxic preconditioning can enhance ISC activation before intestinal insults, such as intestinal transplantation. Therefore, the TLR pathway might be a therapeutic target likely to improve small intestine graft survival [103].

## 9. TLRs Differentially Expressed by Different Stem Cells: Implications for Stem Cell-Based Therapy

From the analyzed data, many different TLR functions emerge in SC, pointing out that SC can have different roles depending on the background and on the kind of ligands that they can recognize. Moreover, we have discussed the TLR involvement in the SC response to a specific tissue damage and in the reparative processes and how the identification of molecules mediating the differential function of TLR signaling could be decisive for the development of new therapeutic strategies. These considerations offer new perspectives for stem cell-based therapy: a pretreatment of the SC with a specific TLR ligand may be conceivable. It could allow a sort of commitment towards cytokine production or else differentiation, for example. On the other side, data on the response of TLR-stimulated cells provides a further element on which to pay attention in order to obtain the success of stem cell therapy. Results on TLRs in immunomodulatory properties of MSC represent a serious warning for the use of MSCs in clinical application. In fact, if on the one hand the immunosuppressive capacity of MSCs represents a key factor for their therapeutic use [32], on the other hand the benefit of using MSCs could be lost when inflammation is present, MSCs can lose their immunosuppressive functions involved in pathogen eradication and in the control of the allogeneic reaction [22]. In this scenario, WJ-MSCs may represent the most attractive tool when immunosuppressive properties are required [24]. Although silencing of some TLR could be a way to maximize the immunosuppressive effect of MSCs, the molecular mechanisms and effects of TLR-priming MSCs need to be still completely understood before paving the way for new immune therapies.

Moreover, we would also highlight that the same TLR could have different effects depending not only on what stimuli it perceives but also on the kind of stem/progenitor cells in which the TLR is expressed and the specificity of the signaling that it can activate. For example, TLR4 triggering can induce very different effects in the different stem/progenitor cells: in MSCs, it induces an immunomodulation increase; in HSC, it induces hematopoietic cell development, in NPC the proliferation and in the hAEC the apoptosis (Figure 1). On the contrary, TLR2 seems to give more similar effects: it induces differentiation in MSC, HSC, NPC, and ARPC, and it induces proliferation and activation with inflammatory response in HSC, ARPC, and hAEC (Figure 1). On the other hand, some TLRs can be expressed specifically in some SCs and can have definite functions that depend on the ligand affinity. As previously reported, TLR5 and TLR6 can bind flagellin and diacylated ligand, respectively, and are expressed in hAECs inducing, when activated, proinflammatory cytokines and metalloproteinases (MMP-9) (Figure 1).

## 10. Conclusion

Considering the available studies on TLRs in SCs, the role and importance of TLRs in sensing an injury by stem/progenitor cells clearly emerges. In some SC types, TLRs can determine their behavior and reparative activity, depending on the conditions in which the cells stand. Therefore, it could be conceivable that SCs employed in therapy could be exposed to TLR ligands, which might modulate their therapeutic potential *in vivo* [31]. TLR agonists are being exploited as vaccine adjuvants for infectious disease or cancer and as therapeutics against tumors. Also, TLR antibodies and inhibitors of TLR signaling pathways have considerable potential as therapeutics for inflammatory disorders [111]. In recent years, some TLR agonists have shown therapeutic potential in different diseases. Imiquimod is a TLR7 agonist with proven antitumor activity as a topical treatment for skin cancer. At the moment, it is approved by US FDA and many phase 2 clinical trials show its safety and efficacy in other types of cancer, such as carcinoma in situ bladder cancer [112], cervical intraepithelial neoplasia [113], or breast cancer cutaneous metastases [114].

Recently, TLR9 agonists have been proposed as a treatment option for glioblastoma (CpG oligonucleotide) [115] or asthma [116], but the phase 2 clinical trials concluded with no additional benefit for patients.

In this context, we need to better understand the mechanisms of action of TLRs on SC and learn how to control these receptors and their downstream pathways in a very precise way, in order to modulate SC proliferation, survival, migration, and differentiation in the pathological environment. In this manner, in the future, cell therapy could be improved and made safer.
